# Elements of Materia Medica and Therapeutics

**Published:** 1845-11

**Authors:** 


### BIBLIOGRAPHICAL NOTICES.

*Elements of Materia Medica and Therapeutics.* By JOHN P. HARRISON, M. D., Professor of Materia Medica and Therapeutics in the Medical College of Ohio. 2 vols. pp. 405, 619. Cincinnati, 1845.

It is not more than twelve months since we gave a running commentary on the chief works that had previously and recently appeared on the subject of Materia Medica and Therapeutics,—a department of medical science less susceptible of rapid modification than any other; for the additions made to Materia Medica in any brief space are few; whilst the great principles of Therapeutics ought most assuredly to be unsusceptible, at this date, of any very material alteration. There appears, indeed, to be a gradually increasing feeling amongst the wisest and the best of the profession, that whilst an exact appreciation and establishment of therapeutical principles is essential, the list of agents for carrying such principles into effect should be pruned, so that ultimately the materia medica may contain only the positively useful. No judicious physician now believes, that there is a single drug which is adapted for the removal of any special morbid condition; whilst all admit the importance of becoming acquainted with the sensible and medical properties of remedial agents; and of applying them, guided by just views of therapeutics.

The work before us is the product of two parturitions. The first volume was ushered into the world several months ago; the latter recently. The author is essentially a vitalist in his views of the *modus operandi* of medicines, which accounts for the remark in his preface, that he feels "in an especial manner" "under great obligations to Professors Chapman, Caldwell and Paine" for his "exposition of the modus operandi of medicines;" whilst he at the same time frankly expresses his acknowledgments to Paris, "Dunglison, Bell, Pereira, and other recent high authorities," for many valuable facts on the questions discussed.

Several of the Author's views in regard to the action of remedial agents are by no means such as we are prepared to embrace. His *first* conclusion, that "medicines acting in accordance with the laws of life, and in a manner coincident to the modes in which impressions producing disease influence the system, do not, as foreign agents, enter the blood; but when found there or in the secretions, they are to be regarded as assimilated, and thus brought under the vital principle"—is by no means demonstrated. Indeed, we should like to hear even a plausible reason offered in its support. What evidences have we of opium, or tobacco, or turpentine, when inhaled, being "assimilated and thus brought under the dominion of the vital principle;" or that any such "assimilation" occurs on the reception of miasmata into the system? And what proofs that saline solutions placed in contact with living vessels, passing into the vessels under the eye of the observer, and capable of being detected in those vessels, have experienced any such change as that indicated by him?

His *second* conclusion we are prepared to receive. "Medicines are relative agents, and therefore never act as specifics;" whilst we unhesitatingly reject his *third*,—that "there is not a single indication of cure, when a true diagnosis of disease is made, which favours in the smallest degree the idea, that medicines, by entering the circulation, accomplish their useful purposes."

Now we affirm that in every case of cachexia, or of any disease, in which we wish to make a new impression on the tissues, the philosophical indication of cure is to administer such agents as may pass into the blood vessels, modify the condition of the circulating fluid, and through it induce a new action on the tissues that are formed and nourished by it. The whole class of eutrophics—as they are termed by Dr. Dunglison—probably exert their powers in this manner,—directly on the blood, indirectly on the tissues. To us, there is a frequent cloudiness, caused by the profusion of words employed by the author to convey his meaning, which induces us to doubt whether we exactly comprehend him, and whether, therefore, we may not be fighting with ideal creations. In other cases, however, his language is sufficiently clear; and in our view—which we by no means hold up as the only true one—sufficiently erroneous. This cloudiness is strikingly apparent in his "*fourth* therapeutical indication," which, he says, "is too much overlooked by the physician"—the "restoration of the secretions"—yet secretions do not become arrested without a pathological cause; and, consequently, the true indication, where arrest of secretion occurs, is to discover that cause, to remove it, and then "restoration of the secretions" will certainly follow.

Still, although we may object to the laying down of any such indication as the "restoration of the secretions," we cannot think the author far wrong, when he cautions us "always to keep in mind, that it is not the quantity of secreted product that is to be viewed as of primary importance, but that the return of the organ to its physiological state, as indicated by the normal secretion, is the criterion of a just sanative effect." vol. 2, p. 420.

We place little stress on classifications of therapeutical agents; and therefore should not object to that of the author, which he says "is founded upon indications of cure," did he not claim for it marked advantages over others. After presenting the subjoined classification he remarks:

"The superiority of this arrangement lies in its easy comprehension, and, as a consequence of its simplicity, a ready reference to the appropriate divisions of the table, containing the separative curative measures in daily use by the physician."—vol. 1, p. 39.

Dr. Harrison's arrangement is as follows:

I. Alterants.—*a.* Anti-inflammatory. *b.* Anti-cachectic or invigorating.

II. Evacuants.—*a.* Bloodletting. *b.* Emetics. *c.* Cathartics.

III. Incitants or Excitants.—*a.* Stimulants. *b.* Antispasmodics. *c.* Tonics. *d.* Astringents.

IV. Secretants.—*a.* Diaphoretics. *b.* Diuretics—Antilithics. *c.* Expectorants. *d.* Emmenagogues. *e.* Anthelmintics.

V. Narcotics, Anodynes.

VI. Derivatives, Revulsives.—*a.* Baths at various temperatures. *b.* Frictions. *c.* Rubefacients. *d.* Epispastics. *e.* Pustulents. *f.* Suppuratives. *g.* Cauterizing counter-irritants.

To almost the whole of this table valid objections may be made. The division of alterants into anti-inflammatory and anti-cachectic or invigorating is far from being precise, and farther still from being clearly demonstrated. The class of Evacuants ought not to exclude Diaphoretics and Diuretics; nor that of Secretants, Cathartics. Antilithics cannot clearly belong to Secretants; nor can we imagine a shadow of a reason for placing Anthelmintics under the same division; nor why "baths at *various temperatures*" should necessarily be derivatives or revulsives! Again, what is the difference between Incitants or Excitants and Stimulants; and by what process of reasoning does the author arrive at the inference, that antispasmodics belong necessarily to the class of Incitants? We cannot, therefore, accord with the author in the confident belief, "that some advancement is made by such an arrangement in achieving a comprehensive and accurate scheme of classification." p. 44.

Our space will not permit us to go into a lengthened examination of the volumes before us. A competent notion may be formed of their contents by the headings of the chapters. In *vol. first* we have, 1. Modus operandi of medicines. 2. Effects of Remedies. 3. Classification of medicines. 4. On the circumstances which modify therapeutical indications. 5. Theory and art of prescribing. 6. Synopsis of incompatible substances. 7. Poisons and their antidotes. 8. Patent medicines and nostrums. 9. Indications of cure. 10. Alterants. 11. Do. 12. Evacuants. The *second volume* is arranged as follows: 1. Bloodletting. 2. Emetics. 3. Cathartics. 4. Enemata. 5. Incitants or Excitants, Stimulants, Antispasmodics, Tonics, Astringents. 6. Restoration of the Secretions, Diaphoretics, Diuretics and Antilithics, Expectorants, Emmenagogues, Anthelmintics. 7. Anodyne or Narcotic Indication; and 8. Revulsive Indication:—a catalogue not characterized for *lucidus ordo*, notwithstanding the fancied "simplicity" of the author's classification.

Dr. Harrison does not confine his accordance with Dr. Chapman to the view of the latter in regard to the *modus operandi* of remedies. He expresses his utter disbelief as to the curability of tuberculosis of the lungs; and in order that there may be no mistake as to his sentiments on this matter, he over and over again repeats his incredulity.

Speaking of iodine inhalations he remarks:—

"That such inhalations may be conducive to the comfort of the patient, we readily concede; but that iodine or any other remedy can exert any restorative power over the tubercular degeneration of the lungs, neither the past nor contemporaneous experience of the profession will justify us in believing, although even as eminent a man as Sir Charles Scudamore should assert that he has cured patients affected with that disease by the administration of iodine inhalations." vol. 1, p. 344.

Under another head his sentiments are not quite so strongly expressed:

"Pulmonary consumption may be prevented, but of its curability strong doubts are entertained by almost every old and observant practitioner, in Europe and America." vol. 2, p. 355.

And again, when treating of Prunus Virginiana:

"As to the curative power confided in by some, which it is said to exercise over phthisis pulmonalis, we agree with Prof. Dunglison, that neither this remedy, and, we may add, no other in our experience, has ever very sensibly checked the progress of tuberculisation in the lungs, when that disorganizing process has been fully established." vol. 2, p. 380.

Notwithstanding, however, the confident assertions of the author as to the incurability of this disease, we consider the fact as well established as any in medicine, that cicatrization of tuberculous cavities does occur, and much more frequently than has been imagined. The modern pathological works contain ample evidence in favour of this assertion.

We shall not say more of the author's style than that it is often turgid, and not unfrequently, we think, violates good taste. This, however, may be regarded as a matter of opinion, on which an honest difference may exist.

"This article" [naphtha) he remarks, "and the one next to be considered [oleum jecoris aselli] are in a point of culmination, whither they have ascended with a rapid progress. Two more certain remedies for that indomitable affection, phthisis pulmonalis, have appeared, and they now blaze in glorious altitude over our heads." And again:

"Naphtha has already commenced a rapid descent from its late zenith ascension, and is doomed to be numbered, ere long, among the multitude of other remedies which have fretted a brief hour upon the stage, and then are heard of no more."

Of naphtha, Dr. Harrison's experience is similar to our own. "In our City Hospital," he says, "and likewise in private practice, we have seen it fairly tested, and the result of our experience of the remedy is, that it has no particular effect on the cure of any serious pulmonic lesion, but that it acts merely as a stimulating expectorant."

The work is printed in good sized type on a page certainly not crowded. Typographical errors, however, are by no means rare, and several of the proper names and technical terms are incorrectly given—as for example:—Thompson, for Thomson, Cuissinier, Lugoll, Bartley for Battley, Guertonian for Guestonian, Mandel for Mandl, Brichatan, for Bricheteau, Bouillard, for Bouillaud, Cruvielhier, for Cruveilhier, Muller for Müller, Liebeg, &c. We may remark also, that we know of no such person as *Prof.* Copland. Bichlormet of Mercury is printed for Bichloruret of M. We observe also, Guiacum,Hæmoptisis, Strichnine, Depillatory, Idiodide of Potassium, Dryobanops for Dryobalanops, &c.

Yet these errors are comparatively venial, when we turn to the formulæ and witness the jumble of languages in which they are written, and the numerous weighty errors. In a work especially intended for the Student, such inaccuracies are altogether inexcusable. Take for example the prescription given to elucidate the theory and art of prescribing.

(Basis) Carb. Ammoniæ, ʒj

(Adjuvant) Gum Camphor, ℈ss.

(Corrigent) Vin. Ipecacuan., ʒij.

(Intermediate) Gum Arab. and Sugar, *aa* ʒij.

Water, ℥vj.

And another from amongst the Formulæ, (p. 73.)

℞ Hydrarg. Protiod. gr. xx.

Lard, ℥iss.

Ol. Bergamot. gtt. xx.

What the following formula means, demands some stretch of the imagination. We have guessed at it, however.

Atropic [Atrophic?] solution.

℞ Potas. Hydriodat. ʒiv.

Ess. Pppt. [?] ʒij.

Syrup, ℥j.

Water, ℥viij.

The absurdity of the formulæ will be more strikingly seen by writing them at length. We take the last.

Recipe.
Potassæ hydriodatis, drachmas quatuor.
Essentiæ menthæ piperitæ, drachmas duas.
Syrup, an ounce.
Water, eight ounces.

Surely, either the Latin or the English should have been exclusively adopted.

